# YouTube Dataset on Mobile Streaming for Internet Traffic Modeling and Streaming Analysis

**DOI:** 10.1038/s41597-022-01418-y

**Published:** 2022-06-13

**Authors:** Frank Loh, Florian Wamser, Fabian Poignée, Stefan Geißler, Tobias Hoßfeld

**Affiliations:** University of Würzburg, Chair of Communication Networks, Würzburg, 97074 Germany

**Keywords:** Computer science, Scientific data

## Abstract

Around 4.9 billion Internet users worldwide watch billions of hours of online video every day. As a result, streaming is by far the predominant type of traffic in communication networks. According to Google statistics, three out of five video views come from mobile devices. Thus, in view of the continuous technological advances in end devices and increasing mobile use, datasets for mobile streaming are indispensable in research but only sparsely dealt with in literature so far. With this public dataset, we provide 1,081 hours of time-synchronous video measurements at network, transport, and application layer with the native YouTube streaming client on mobile devices. The dataset includes 80 network scenarios with 171 different individual bandwidth settings measured in 5,181 runs with limited bandwidth, 1,939 runs with emulated 3 G/4 G traces, and 4,022 runs with pre-defined bandwidth changes. This corresponds to 332 GB video payload. We present the most relevant quality indicators for scientific use, i.e., initial playback delay, streaming video quality, adaptive video quality changes, video rebuffering events, and streaming phases.

## Abstract (summary)

**Table:** 

Measurement(s)	Mobile video streaming with YouTube
Technology Type(s)	Tcpdump and publicly available wrapper app for YouTube data
Sample Characteristic - Environment	mobile phone • YouTube app
Sample Characteristic - Location	Germany • Europe

## Background & Summary

The nationwide rollout of new mobile communication technologies requires in-depth traffic analysis, usage studies, and network management. With more than 4.9 billion Internet users worldwide^1,2^, online videos and entertainment are among the most popular activities of users, and thus, of particular interest. However, datasets on Internet traffic dealing with mobile streaming of videos from major streaming platforms are currently sparsely treated in literature. Thus, the study of current mobile traffic and prediction of future traffic for accurate network management is a challenging task without a data basis.

Overall, approximately 1.24 billion monthly users watch nearly 1 billion hours of online video on YouTube every day^3–5^. Statistically speaking, every person in the world watches an average of 7.55 minutes on YouTube every day^5^. This makes YouTube the second most visited website in the world^6,7^, with mobile access accounting for two-thirds of the platform’s video views in the second quarter of 2021^8–11^. In fact, YouTube generates over a fifth (20.4% downlink, 5.4% upstream) of all global mobile Internet traffic^12^. But in literature are only a few datasets available that document the use of YouTube in mobile environments. The available archives mainly concentrate exclusively on data collection via the desktop version of YouTube^13–15^.

The desktop version used in modern web browsers behaves differently from Android or iOS versions, as different libraries, application types, and operating systems come into play^13,16,17^. Thus, it is not representative for mobile usage. Although YouTube follows the Dynamic Adaptive Streaming over HTTP (DASH) standard^18^ on both platforms, it uses different settings for adaptive streaming^16^. Ramos *et al*.^19^ show that, for example, other buffer threshold values are used for the mobile app. Furthermore, YouTube mobile is more aggressive with throttling factors at higher encoding rates. Other studies show a far greater use of the Quick UDP Internet Connections (QUIC) protocol^20^ for mobile applications than for desktops^21^. The few public datasets on YouTube that contain measurements from mobile clients^16,19,21,22^ provide network *or* application traces only. Focusing solely on one type of trace offers only a limited view of the streaming process, since there are interactions between the network and the application, especially during adaptive video streaming.

Our dataset^23^ aims to close this gap by providing measurements that were obtained simultaneously at the network, transport, and application level. The data was generated using YouTube’s native Android application over 29 months between January 2018 and May 2020. We provide 1,081.18 hours of time-synchronized video measurements, resulting in 45 days of continuous video with the native YouTube streaming app on mobile devices. The dataset includes 11,142 measurement runs conducted with 171 different bandwidth limitations used in 80 different network scenarios. The measured data corresponds to a total of 332 GB of video payload with TCP and UDP/QUIC traffic. Thus, this dataset stands out from related work in particular with the combination of a large number of different network scenarios and videos to understand, model, or predict current or future networks.

At the application, we extracted a wide range of adaptive streaming parameters from YouTube’s mobile client, among others, streaming quality, buffer level, and frame playout information. We recorded available and generally accepted parameters in the network such as packet length, number of flows, transport protocol, and transport layer protocol ports. This was made possible by our freely available wrapper app^24^, which enables remote control and monitoring of the native Android application by YouTube. The dataset was extensively post-processed and annotated. We present the most relevant quality indicators for scientific use, namely initial playback delay, streaming video quality, adaptive video quality changes, and video rebuffering events.

The idea behind the dataset is on synchronous measurements at the network, transport, and application layer in strictly controlled environments. The main goal of the measurement setup is to ensure to replicate real world circumstances as closely as possible for a mobile user. This allows for the comparison between network and application information. The measurement environment follows the guidelines of the DASH Industry Forum^25^ to enable controlled measurements so that throughput, packet error rate, and streaming quality can be controlled during the measurements. Thus, the mobile use can be documented as comprehensively as possible with a focus on application-layer and quality indicators, in addition to the technical network and streaming parameters.

## Related Work

Datasets regarding applications of video streaming as well as adjacent areas have been published in recent years. To integrate this work in the broad landscape of related work, the following section and Table 1 provides an overview of selected works.

**Table 1:** Overview of selected related datasets.

reference	video measurements	application	data	data focus	dataset size
Barman ’18^37^	✗	Gaming	uncompressed video data	research on gaming video quality assessment	24 videos, 30 s each
Nguyen ’19^42^	✗	360° video	saliency dataset for 360° videos	attention models, head movement prediction and video tile preparation for 360° videos	24 videos, 50,654 saliency maps
Lall ’21^29^	✗	Netflix	viewing activity data	group users based on activity level, get watch patterns, user preferences etc.	1,060 users, 1.7 M episodes and movies
Zabrovskiy ’18^30^	✗	DASH videos	multi codec DASH (AVC, HEVC, VP9, AV1)	videos encoded in different formats for streaming experiments	10 videos, 19 bitrates, 4 codecs
Baccour ’20^34^	✓	Facebook Live	video & metadata	data overview of facebook live videos, viewers, broadcasters	1.5 M live streams
Sengupta ’15^22^	✓	YouTube native app	network traffic	Smartphone app traffic traces collected using tcpdump	3 GB trace
Karagkioules ’18^21^	✓	YouTube native app	application & network	provide test cases for YouTube’s adaptive streaming logic	374 h, 3 videos
This work	✓	YouTube native app	application & network & transport	raw data for model creation, machine learning, quality prediction	1,081.18 h, 246 videos

In general, datasets exist for audiovisual and subjective measurements and studies uploaded at the Qualinet database^26^. The datasets include, among others, DASH, H264 and H265, mobile video quality, or QoE datasets. For video streaming only, and in particular, several datasets already exist for the YouTube platform dealing with watch histories^27^, video application information, key-frame distribution and object names for search engines^28^. For viewing activity, in particular, Lall *et al*. published a dataset recently for Netflix^29^. It includes 1060 users and more than 1.7 M watched episodes and movies. However, none of these works take the video itself into consideration.

This is done for example by Zabrovskiy *et al*. in 2018 for DASH videos^30^. The authors present a dataset with multi codec DASH videos for ten different videos, 19 bitrates and four codecs. Other works and datasets focus on video segmentation information^31,32^. Furthermore, Wang *et al*. studies YouTube’s user generated content for video compression research and published a dataset for that purpose in 2019^33^. In addition, real streaming data and video meta-data is discussed by Baccour *et al*. for Facebook Live^34^. The authors published a data overview of Facebook live videos, viewers, and broadcasters of more than 1.5 M live streams. For YouTube streaming in particular, datasets exist for very specific metrics like the initial delay^35^ or aggregated application and network data to be used in machine learning and for quality prediction^36^.

Back in 2011, Rao *et al*. published an initial mobile web browser and native app dataset for YouTube^16^ and Alcock and Nelson studied the application flow control in YouTube video streams^13^. These works were extended in 2015 with a raw network trace dataset of measurements with the mobile YouTube app in 2015^22^.

More recently, Karagkioules *et al*. published a small dataset with application and network data for the native YouTube app with three different videos and eight different bandwidth and quality settings in 2018^21^. However, because of the small video and network setting diversity, the usability for streaming quality prediction or streaming modeling is limited. Thus, in general, recent and sufficiently large raw full packet trace information or minimally processed data is missing to date.

In contrast, in the dataset presented in this work, the full network packet trace containing network and transport layer information is available together with all application data, and thus, the complete streaming behavior. In total 246 different videos are included with 171 different individual bandwidth limitations and more than 1,000 h of total video playtime. In particular, the large number of different videos and bandwidth limitations is, to the best of our knowledge, not available so far in literature.

The importance of datasets is increasing in many application areas. In recent years, gaming is also becoming more and more relevant in the context of multimedia and video data transmission. Barman *et al*. published an initial dataset with uncompressed video data to study gaming video quality in 2018^37^. This work has recently been extended by Zhao *et al*.^38^. In their work, the authors provide a test dataset of gaming video content together with a performance analysis of existing coding tools. Besides gaming, 360° video is one hot topic in recent multimedia data transmission research. There, several works with interesting datasets study content and sensor data^39^, head movement^40^, and head together with eye movement^41^ to optimize 360° videos or reduce traffic requirements based on different metrics. In addition, Nguyen and Yan presented a saliency dataset for 360° videos in 2019^42^. The goal of their dataset is to give other researchers the opportunity to create attention models, head movement predictions, or video tile preparations for 360° videos based on 24 videos and more than 50,000 saliency maps.

## Methods

The procedure for creating our dataset is based on the design and deployment of a testbed and the definition of the used measurement procedure. In addition, post-processing steps to enrich the dataset with additional streaming information is presented in this section. Both, the raw data and the post-processed information is included in the published dataset.

### Testbed description

**Fig. 1:**
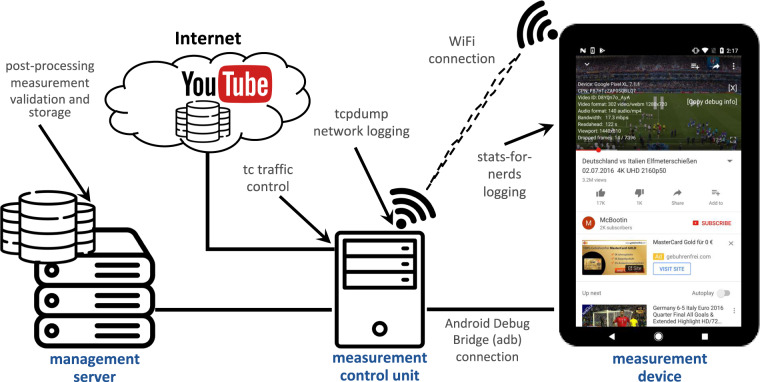
Tested overview.

To ensure that real-world scenarios are replicated as precisely as possible, a client-server-based measurement setup was developed to record both network and application data. The full setup is presented in Fig. 1.

The management server is an entity that validates and organizes measurements and does not take an active part in the measurement process. Instead, its responsibilities include the validation, post-processing, and storage of data. Examples for validation steps are scans for empty or erroneous files, the correct enforcement of bandwidth limitations, and extensive logging of events occurring during the measurement process.

The measurement control unit is responsible for the actual measurement process and is connected to the management server. It is equipped with a sufficiently powerful processor, an i7-4770 processor with 8 × 3.40 GHz and 16 GB RAM to avoid bottlenecks during the actual measurements. It is connected to the Internet via the German national research and education network (Deutsches Forschungsnetz) via fixed connection to guarantee that measurements are not impacted by physical bandwidth limitations. To control uplink and downlink bandwidths, the state-of-the-art Linux command line tool *tc*^43^ is used for traffic shaping and control in the Linux kernel. To perform actions, such as starting measurements or logging application data, the measurement control unit is connected to the measurement device, a smartphone, via the Android Debug Bridge (adb)^44^. This way can be used to directly connect to Android devices via USB and does not interfere with measurements. Finally, the measurement control unit provides a wireless connection with variable and controlled QoS parameters for the measurement device itself. The interface of the wireless access point is the monitoring point for the complete network traffic containing network and transport layer data. All upstream and downstream traffic is captured using *tcpdump*^45^ within the Linux kernel ringbuffer between the wireless network interface and the connected device. Data is stored locally on the measurement control unit before it is offloaded to the management server for further processing.

The measurement device is connected to the Internet via the provided 2.4 GHz WiFi access point. To exclude unintended bottlenecks at the smartphone, for the measurement setup, a clean, new Google Pixel XL with Android SDK version 28 released in 2020 is used. The display resolution of 2560 × 1440 pixel (equal to the 1440p resolution of YouTube) does not limit the video playback, and the 2.15 GHz and 1.6 GHz quad-core Qualcomm Snapdragon 821 processor and 4 GB RAM are sufficient for the playback of videos. Please note that playback related decisions are triggered by the YouTube app and not by the phone. Thus, it is not expected to achieve other results with other Android-based smartphones if the available resources are sufficient. An in-depth study of the performance with Apple’s iOS is subject of future work. However, we expect no large differences. Furthermore, no additional applications are running at the smartphone and the battery is kept at sufficient health during all measurements. The chosen app and OS version is available in the dataset. During a measurement run, a video is played and relevant application layer metrics are logged directly by the device. To achieve this, a specially developed wrapper app has been developed to monitor the native Android YouTube app exactly as it is distributed through Google Play Store. The source code of our tool is freely available on Github^24^. Seufert *et al*.^17^ have published a detailed description of this wrapper app and the measurement process used in this work.

### Measurement description

The steps of a single measurement run are defined as follows. Every measurement is started by the measurement unit. In a first step it checks for available connections to the management server, the Internet, and the measurement device via adb. Upon success, a WiFi access point is opened to provide Internet connectivity to the measurement device. Subsequently, the network scenario is defined. Either no bandwidth limitation can be set for the complete measurement, which results in approximately 400 Mbit/s downlink bandwidth (and thus no impairment when streaming videos between 144p and 1080p) or a predefined bandwidth setting schedule can be used. In the latter case, bandwidth limitations are planned for the complete measurement by dynamically applying different limits based on either synthetic traffic limitations or real-world bandwidth measurements of 3 G and 4 G mobile networks. Afterwards, prior to the actual playback of the desired video, a *setup video* (ID FiO0iLzTyVg) is played for 10 s. This is done to ensure that all network, transport, and application data of the desired video can be logged and the bandwidth setting is applied correctly. During this *setup video*, the player can adapt the requested playback quality towards the initial bandwidth setting. This avoids unwanted playback behavior that is not a result of the defined scenario but of the switch to the initial bandwidth limitation. Afterwards, a YouTube video is selected for the measurement based on a predefined list of video URLs.

Beginning with the measurement start, after connectivity to all components has been established, all network and transport data transmitted and received at the WiFi access point is logged. This includes especially the uplink and downlink video data from the measurement device. Furthermore, as already mentioned during the testbed description, the application data is logged directly at the measurement device by parsing and storing the *stats-for-nerds* data provided by the native YouTube app once a second. In these *stats-for-nerds* information, the complete application behavior like buffer filling status, played video, or the number of already played frames is logged. This data is transmitted immediately to the measurement control unit and written to a file via the USB-connection to not interfere with the WiFi connection used for the measurement. After video playback is finished, all network, transport, and application data points are sent from the measurement control unit to the management server for validation and further post-processing steps. The network and transport data include the timestamp, the source and destination IP address, source and destination port as well as packet lengths for all TCP and UDP packets observed during the measurement period. The application data include a timestamp, the currently played out video and audio quality, the frames per second, buffer status information, the number of dropped and already played out frames as well as the video ID. Additional information include the current App version, OS version, number of connections, and the battery status during the measurement. An overview of all logged network and application layer metrics is summarized in Table 2.

**Table 2:** Measured parameter overview.

Application data parameter	Explanation	Example log
timestamp	timestamp of logged information	2020-02-13 22:39:23.001
fmt	video stream format code^59^	247 (equal to 720p)
fps	frames per second	25
afmt	audio format code	140 for 128k m4a audio
bh	current buffer filling level in milliseconds	39939 for 39.939 s
droppedFrames	number dropped frames	0 (equal to no dropped frames)
playedFrames	number played out frames	1258
videoid	current played video ID	6fd2kLmSDQ
cbrver	YouTube app version	14.46.52
cver	YouTube app version	14.46.52
cosver	OS version of smartphone	9
conn	number of parallel open connections	3
bat	current battery filling status	1.000:1 (equal to full battery)
**Network data parameter**		
timestamp	epoch timestamp of packet arrival	1581633565.013519
ipSrc	source IP address	10.10.0.140 (device IP in own network)
ipDst	destination IP address	74.125.13.143 (Google server IP)
tcpPortSource	TCP source port	443 (as HTTPS port)
tcpPortDst	TCP destination port	37475
udpPortSrc	UDP source port	443 (only available if UDP traffic)
udpPortDst	UDP destination port	48372 (only available if UDP traffic)
tcpLen	TCP segment length in Byte	1358 (payload length in Byte)
udpLen	UDP packet length in Byte	1358 (payload length in Byte)
payloadProtocolNumber	used transmission protocol (TCP or UDP)	6 (for TCP), 17 (for UDP)

### Data post-processing

**Fig. 2:**
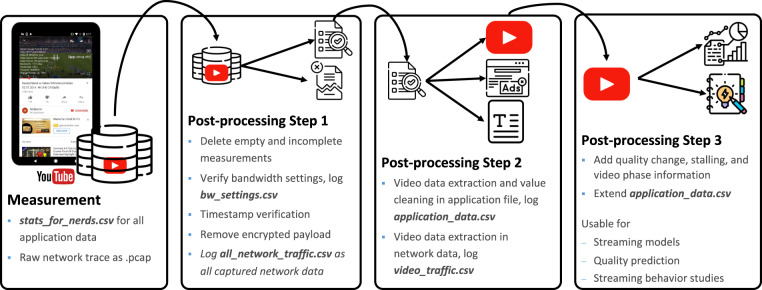
Step by step post-processing after measurement.

The post-processing of the data presented in this work is done by three major steps according to the overview in Fig. 2. All files that are added to the dataset in each specific step are highlighted in the figure. After the measurement is completed, erroneous, invalid, or partly missing measurement data is discarded in the first post-processing step to ensure a complete, high quality dataset. The remaining application, network, and transport measurements, enriched with information regarding the bandwidth settings during the measurements are included in the dataset.

Second, to simplify a streaming behavior study, flows related to YouTube streaming are identified based on their IP-addresses. Application data is cleaned by removing advertising data points related to the playback of commercials. This *cleaned* data is the second part of the dataset.

In the last post-processing step, data is processed to further simplify usage and extend the usage potential. In this last step, streaming phases are defined describing the current *player health* which means whether the video player receives enough video data to fill or keep the current playback buffer health or if the buffer fill level is declining. This additional information is included in the third part of the dataset. To receive a more general idea about the post-processing in this work, these three steps are explained in detail in the following.

#### Post-processing Step 1: Data integrity check and data cleaning

In the data integrity and cleaning step, all invalid measurements are discarded. Therefore, the following tests are performed.

##### Empty or incomplete measurements

First, all measurements where either the raw network file or the application stats-for-nerds file does not contain data are deleted. This may occur if the YouTube app was not opened correctly during measurement pre-start. Furthermore, inconsistent measurement runs for which network traffic is measured longer than application data or vice versa are deleted. This occurs if the *tcpdump* capture crashes or if the application data logging is faulty.

##### Correct bandwidth setting

In this step, it is determined whether the bandwidth was set correctly. Therefore, bandwidth limit changes logged during the measurement process by the testbed are compared with the predefined bandwidth setting. Furthermore, all network data are analyzed to determine if the network throughput exceeded the possible bandwidth limitation. All error-prone measurements are discarded. For the remaining measurements, the bandwidth information is logged with the timestamp and the set bandwidth limitation in the *bw_settings.csv*.

##### Timestamp verification

The timestamps of all measurements are checked for plausibility. Since both sources, network and application data, contain timestamps which are supposed to give a complete view together for the measurement duration, the overlay of the timestamps is validated. There is no exact congruence because the network layer first establishes a connection and starts downloading video data before the application logs are filled. Similarly, no more data is downloaded at the end of the video when the rest of the content is already in the buffer, but the application logs still show video playback.

#### Encrypted payload removal

Since the raw network data from the packet capture file contains the encrypted payload from which no further information can be extracted this data is further processed. By means of tshark^46^, relevant network and transport layer traffic information listed in Table 2 is extracted and included as *all_network_traffic.csv* in the dataset. All raw application data presented in Table 2 is received after this step with a 1 s to 3 s granularity, depending on the current player status or on player issues. Player issues can occur if not enough data is downloaded because of, for example, very little bandwidth during measurements. Then, the player crashes or error-prone values are displayed and logged. All application data is included in the dataset as *stats_for_nerds.csv*.

#### Post-processing Step 2: YouTube streaming data extraction

In the second post-processing step, relevant video streaming data is separated from cross-traffic. Furthermore, error-prone values in the application *stats-for-nerds* file are cleaned.

##### Application data video extraction

The video data extraction is simple for the application data since the video ID is available for each measurement point. Thus, only the application information for the measured video ID is kept while other video IDs, especially the *setup video* described earlier as well as advertising played during the measurement process is excluded. Since this dataset presents YouTube streaming data, all measurement runs where the correct video is never played because of too long advertising are discarded.

##### Value cleaning: buffer health

In this step, all values in the *stats-for-nerds* logs are checked for valid ranges. During this process, negative buffer health values are found frequently in the proximity of a video quality change. Via manual validation in the application, this inconsistency is confirmed to be a logging issue of the YouTube application itself. If the quality of a video changes, the buffer health level may change suddenly since the adaptive streaming algorithm switches the input for its buffer health prediction from the buffered content in the previous quality to the buffered content in the upcoming quality. This can lead to very low or even negative buffer health values in the logs. However, these negative values occur regardless of changing to a higher or lower video quality. For example, the playback may be uninterrupted, and quality changes to a higher level. The negative buffer health values occur when the client decides to request a new quality, but neither has the client changed to the new quality nor downloaded enough content in this quality to allow for a quality change without playback interruption. After this step, all valid data points from the *stats-for-nerds* files without advertising and setup video are added as *application_data.csv* to the dataset.

##### Network data video extraction

For the network and transport data, there are two possible ways of separating video data from cross-traffic. For many measurement runs, it is possible to extract the video flows by following IP-port-tuples based on the DNS resolution for *googlevideo.com*^36^. These flows are identified as video flows and separated from other traffic. However, to filter not only cross traffic, but also the traffic of the *setup video* at the beginning of each measurement run and specific advertisements, another approach is used in addition. Firstly, from the application data information the start and end time of the correct video is determined for each measurement run. Next, from the network data, all flows which are active within that time window are considered candidates for the video stream. Candidate flows are marked and listed with the complete traffic in descending order by traffic volume. Afterwards, the candidate flows are added as streaming flows to the dataset until the traffic volume accumulates at least 90% of the total traffic during the video stream. This is valid since video flows are identified as dominant flows in YouTube streaming measurements^47^. With this method, cross-traffic like loading of video comments or video recommendations, transmission of DNS requests, and other background processes are excluded. Furthermore, including only the largest flow would not be sufficient because YouTube may change the connection to a different server during a video stream in case of, for example, data transmission issues, quality changes, or video rebuffering. The remaining traffic is added as *video_traffic.csv* to the dataset.

#### Post-processing Step 3: Dataset extension

In the third and final post-processing step, the dataset is extended by critical quality metrics during video streaming that are not included so far: quality changes and stallings (i.e., video rebuffering events)^48^. Please note, information about the start up delay and an explanation of the calculation procedure with our dataset is already published in the work^35^ and thus not included here. Furthermore, the streaming phase indication is introduced that describes whether the player is currently in good condition and receives enough data to constantly keep the buffer filled or not. Therefore, the *application_data.csv* file is enriched with additional information. The *stalling* column indicates that the playback of the stream is interrupted because of a buffer underrun. Since playback is never paused during the measurements, this information is achieved by comparing consecutive *playedFrames* values. If no frames are played out between two log entries, the stream is assumed to stall.

The columns *qc* and *qcTo* are added to list all quality changes. If the *fmt* value in the application data log changes from the current to the next timestamp, the video quality changed. Then, the *qc* value is set to 1 and the *qcTo* value is set to the target video quality code.

Moreover, we provide an estimate about the current video phase based on a video phase detection algorithm. Each video is in one of four phases during an ongoing streaming session: *filling* if the available playback time in the buffer is filling, *steady* if the buffer level remains constant, *depletion* if it decreases or *stalling* if no frames can be played out because of a buffer underrun.

All values in the first and last 5 s are assigned as *filling* and *depletion* respectively, since in the beginning and the end of playback the video is always in these phases. Afterwards, all other logs with a buffer level below 1.2 s are set to *stalling*. This shows good results in practice since the player can only play out completely downloaded video segments. For that reason, in most cases some playback time is left and the buffer does not drop to zero. The threshold of 1.2 s was chosen by looking at the maximum buffer level during a stalling event in the data. The remaining logs are listed as *steady* if the buffer level does not change more than 0.3 s between two logged values and the overall buffer is larger than 5 s. We choose the slope boundary of 0.3 s by looking at the occurring slopes in unlimited bandwidth scenarios where a steady phase can be determined manually. This ensures that small changes in the buffer health do not prevent the algorithm from detecting a steady phase and too small buffer levels are not set to *steady* since a buffer health level of less than 5 s is not enough to guarantee a smooth video playback experience if the bandwidth fluctuates. All other values are set to *filling* if the buffer level is increasing or *depletion* if it is decreasing. Please note: if the quality of a video changes, the buffer health level changes suddenly based on the pre-loaded data for the upcoming video quality. This can lead to small or even negative buffer health values in the logs as described in the data cleaning above. This is a logging inconsistency as the drop may occur before the video quality is changed for the user. To correct false assignments of a stalling phase in this case, it is checked whether it is possible to have played out all the pre-loaded data from the previously observed log value. If this is not the case, the value is set to the previously selected phase value.

After this correction, the phase detection values are smoothed to prevent frequent jumps out of and back into steady state. The minimum duration of a steady phase is 15 s. If within 10 s after a steady phase another data point is labeled as *steady* the entire period is set to *steady*. Furthermore, the same procedure is applied for short jumps out of the stalling phase. This is in accordance with Gutterman *et al*.^49^. A technical description of the phase detection algorithm is out of scope at this point. Instead, a detailed description by a pseudocode is included in the dataset.

## Data Records

The dataset presented in this work contains 11,142 individual video measurements^23^. Each measurement contains five files as described above. The complete network trace with all network and transport layer information is available in the *all_network_traffic.csv* file in each folder, the extracted video traffic captured from the network is available in the *video_traffic.csv*. The complete application log, as it is available in the stats-for-nerds information directly from the YouTube app, is logged in the *stats_for_nerds.csv*, while the extracted video-only application data together with the extended dataset information as described in post-processing step 3 is available in the *application_data.csv*. To provide information about the network conditions during each measurement, the *bw_setting.csv* lists the network bandwidth limitation at specific timestamps for each measurement. If the available bandwidth is changed during the measurement, this bandwidth is kept until another change is performed and logged in the file. Furthermore, the data records include a pseudocode of the phase detection algorithm, several evaluation and visualization scripts, a large general overview csv file, and the post-processing scripts to process own measurements similarly to this dataset and reproduce each step for interested readers. In the following, detailed statistics about the data records in general are provided followed by observed details and special characteristics during different bandwidth limitation settings.

### General data overview

The general data overview is split in three main categories: application, network, and bandwidth information as summarized in Table 3. The total dataset of 11,142 measurement runs contains 246 different videos with a total playtime of 1081.18 h and more than 100 M played frames. Please find a video catalog file (*video_catalog.csv*) in our materials folder. This file includes all video IDs, duration, available resolutions per video, fps, and the video genres. The application data contain 19,242 total quality changes in 6,929 different measurement runs, 35,558 total dropped frames, and 8,652 total stallings in 3,734 different measurement runs. A total video payload of 332.75 GB is downloaded contained in close to 375 M video packets. The dataset contains 242,973 total video flows while 1,288 measurement runs contain only TCP traffic as transport protocol and 8,996 contain only QUIC traffic logged as UDP in the dataset. The remaining 858 contain both, TCP and QUIC traffic.

**Table 3:** General dataset overview.

Application		Network		Bandwidth	
total measurement runs	11,142	downloaded video payload	332.75 GB	max bandwidth	400,000 kbit/s
different measured videos	246	total video packets	372,945,168	min bandwidth	17 kbit/s
total video playtime	1,081.18 h	total video flows	242,973	total bandwidth changes	92,508
total played frames	100,543,176	TCP only traffic runs	1,288	measurements const. bandwidths	5,181
total dropped frames	35,558	UDP only traffic runs	8,996	measurements planned bandwidths	4,022
total quality changes	19,242	TCP & UDP runs	858	measurements real bandwidths	1,939
total stallings	8,652				

**Fig. 3:**
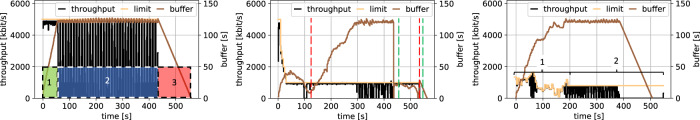
Visualization of throughput and buffer filling status for each bandwidth scenario as an example: left shows constant bandwidth limits with different streaming phases (1 - filling phase marked in green, 2 - steady phase marked in blue, 3 - depletion phase marked in red); middle shows variable pre-defined bandwidth setting (quality changes to lower quality marked by red line and to higher quality in green); right shows 3 G bandwidth trace for first 200 s (1) and a constant bandwidth limit afterwards (2).

The complete dataset is measured with 171 different individual bandwidth limitations used in 80 different network scenarios. A general overview of all bandwidth scenarios is included in the *bwlist.txt* file in the dataset. The maximal bandwidth is 400 Mbit/s which is the maximal possible network bandwidth in the university network in Würzburg, Germany. The minimum is set to 17 kbit/s from a 3 G bandwidth trace. A total of 92,508 bandwidth changes measured during video playback. The bandwidth limitation scenarios can be split in three categories: 5,181 measurement runs are conducted with *constant bandwidth limits* during the complete measurement run, 4,022 runs have *pre-planned bandwidth settings* that include: incrementally increasing or decreasing bandwidth in predefined time intervals, abrupt bandwidth drops to trigger quality changes or stalling, and fluctuating bandwidth settings between specific predefined settings. The remaining 1,939 measurement runs are conducted with *emulated real bandwidth traces* received from real 3 G^50^ or 4 G^51^ network traces. In these measurements, we update the bandwidth limitation every 5 s to a new value from one network trace. We have chosen the bandwidth settings to study three main situations during the YouTube streaming: (1) understanding the streaming procedure in general, (2) gaining knowledge about scenarios with playback issues and limited bandwidth, and (3) study the streaming process under conditions as similar as possible to reality. For that reason, we have chosen the bandwidth settings as follows: For our first goal, we selected constant bandwidth settings to monitor streaming in very regular, and for high bandwidth limitations, good conditions. With these settings, one can understand the streaming process in general and get many baseline details for streaming that helps, for example, in streaming issue prediction. Furthermore, we increased the steps of bandwidth limitations for larger limits since more than 10 Mbit/s was usually sufficient for a good streaming experience. For lower bandwidth limitations, smaller changes affected the playback behavior more severe and was thus, measured in smaller steps. As a second goal, we wanted to generate playback issues for the app. Therefore, we tried to trigger video re-buffering events with abruptly changing bandwidth to achieve a better understanding of this condition. With more slowly changing bandwidth settings, we wanted to trigger among others, quality changes, buffer level changes, or in general changing conditions in the app. Last, the goal was to test the behavior in realistic conditions with the emulated 3 G and 4 G scenarios. This helped us to understand whether stalling or varying playback quality is really an issue in real networks. This understanding can help to react on decreasing buffer situations early and improve the buffering behavior in general to avoid stalling and increase the user perceived quality. In the following, one example measurement for each of the bandwidth setting options is presented with additional information. A visualization is presented in Fig. 3. All plots are structured as follows: the left y-axis shows the throughput in kilobit per second with the set throughput limit during the measurement indicated by the yellow line and the actual measured data throughput in black. The x-axis shows the timeline from video playback start to video playback end. The right y-axis shows the buffer filling status in seconds with the buffer level plotted by the brown line. Additionally, different further information is added to each plot that will be discussed in detail in the following.

#### Constant bandwidth limitations

In the dataset, 5,181 video runs were measured with constant bandwidth limitations. In these scenarios, one bandwidth limitation is set and kept for the complete measurement run. The constant bandwidth limitations include 0.2 Mbit/s up to 2.0 Mbit/s in steps of 0.1 Mbit/s, 2.0 Mbit/s up to 3.0 Mbit/s in steps of 0.2 Mbit/s, 3.0 up to 6.5 Mbit/s in steps of 0.5 Mbit/s, and 1,024 kbit/s, 7.0 Mbit/s, 8.0 Mbit/s, 9.0 Mbit/s, 25.0 Mbit/s, and 400.0 Mbit/s. The results of these measurements show 5,533 quality changes that is equal to 1.07 per measurement run on average at application layer. Since in the complete dataset 1.73 quality changes per measurement run are detected on average, the quality change probability is lower. Furthermore, like expected, fewer quality changes are detected with increasing bandwidth limitations. For all measurements with bandwidth limitations larger than 1.0 Mbit/s, only 0.677 quality changes on average per measurement run are detected, for measurements with more than 5.0 Mbit/s, it is only 0.210. Similar data is measured for stalling occurrences. A total of 1,328 stallings are measured in all constant bandwidth limitation scenarios. Thus, on average 0.256 stallings are detected per video measurement run with constant bandwidth limitations compared to 0.776 for the complete dataset. With increasing bandwidth, again like expected, the average number of stallings per measurement run is decreasing with only 0.073 on average for measurements with more than 1.0 Mbit/s.

For that reason, these scenarios are valuable to study and understand the general streaming behavior without the influence of bandwidth fluctuations. Especially the downloading and buffering behavior is included and can be studied in detail. This is shown in the left subfigure of Fig. 3. The figure shows an example measurement run with a constant bandwidth limitation of 5 Mbit/s for video ID *N2sCbtodGMI*. The video playback starts with a fast and constant buffer filling in the first 55 s shown by the brown line. There, the complete throughput limit is used shown by the black line that is constantly close to the limit. This part is labeled with (1) in green as filling phase. In this phase, more data are downloaded than played out and the buffer level is increasing. Afterwards, in the steady phase (2) marked in blue, the buffer level is kept at a constant level between 120 s and 125 s. The complete bandwidth is not required in this phase shown by the throughput spikes up to the bandwidth limit. At the end of the video, the buffer level is decreasing in the depletion phase (3) shown in red. In this case, the complete video is already downloaded and no more data is required since no download at all is detected. However, such depletion phases are also detected if the available bandwidth is lower than the required throughput to download the current video playback quality.

#### Variable predefined bandwidth settings

To study the buffer depletion phases in more detail and especially the resulting potential quality changes or video stalling events, the bandwidth limit must not be kept at the same single level. Thus, 40,022 video streams are measured with 20 pre-planned, fluctuating bandwidth settings to trigger these scenarios.In these bandwidth scenarios, the bandwidth limit changes from for example 800 kbit/s, 1.0 Mbit/s, 3.0 Mbit/s or 5.0 Mbit/s down to 1.0 Mbit/s or less in several steps (please find all bandwidth settings as an overview in the *bwlist.txt* in the dataset). After the bandwidth dropped, it either keeps at a low level or it increases again. The resulting data from these scenarios include 10,028 total quality changes or an average of 2.493 per video and 6,689 total stallings which is equal to 1.663 per measurement run on average. Furthermore, more than 35,000 frames are recorded as dropped for all measurement runs with variable predefined bandwidth settings which suggests issues during playback, buffering, or video download.

The measured bandwidth changes result in download rates lower than the currently played out video bitrate and are used to study the buffering, and especially buffer depletion phases that lead to quality changes. Furthermore, it is possible to investigate stalling avoidance mechanisms of YouTube mobile streaming. An example scenario for a measurement of video ID *2d1CVrCvdzbY* is shown in the middle plot of Fig. 3. In this measurement, the starting bandwidth is set to 5.0 Mbit/s for the first 10 s. After 10 s it changes to 3.0 Mbit/s and then it drops additional 500 kbit/s each 5 s down to 1.0 Mbit/s. This limit is afterwards kept until the end of the measurement.

The influence of this behavior is visible in the buffer level progress. After a fast increase at the beginning of the measurement, the increase is slowed down with the bandwidth drops. At a bandwidth limit of 1.5 Mbit/s, the increase is stopped and the buffer starts to decrease slowly after the drop to 1.0 Mbit/s. At 100 s playtime, a drop in the downlink bandwidth is detected which is assumed to occur due to a quality change triggered at network layer. This quality change from 720p to 360p is afterwards detected at 130 s marked by the red dashed line. It is assumed that the already pre-buffered old quality is played out before the actual quality change is visible in the application data information. Afterwards, the buffer is filling again up to 120 s at 300 s measurement time. At 437 s, a large drop in the buffer filling level is detected while in addition directly afterwards, the buffer is filling again. We assume that this behavior is another quality change triggered at network layer. The buffer level is already updated to the new quality in the application file but the quality information keeps the old quality. This is changed at 460 s, where the actual quality change from 360p to 720p is performed shown by the green dashed line. Please note that in this case, at 437 s, 120 s video is pre-buffered for quality 360p. With the quality change to 720p at 460 s, only 23 s of the remaining video is played out and much data is discarded. Afterwards, since the bandwidth is still not sufficient to keep the buffer at a constant level for 720p quality, another quality change down to 360p is triggered at 534 s and again up to 720p at 555 s.

#### Emulated real bandwidth trace settings

Since a constant bandwidth limit or pre-planned fluctuating bandwidth limitations are inappropriate to study the streaming behavior in real mobile networks, 1,939 measurement runs are conducted with real 3 G and 4 G bandwidth traces. The current bandwidth limitation is updated according to values from real 3 G and 4 G traces every 5 s as trade-off between very frequent bandwidth changes leading to possible computational or update overhead and sufficient accuracy. Smaller values are closer to real bandwidth settings while larger values smooth unwanted behavior or measurement errors more. To compare the behavior during real bandwidth scenarios and constant bandwidth limitations for the same video at the same time, the bandwidth from the traces is applied at the beginning of the measurements while, for example, after 200 s, a constant bandwidth limit is set. During these measurements, 3,681 total quality changes or 1.898 quality changes on average per measurement run are detected. Thus, it is shown that real bandwidth scenarios also trigger many quality changes. However, only 635 stallings that is equal to 0.327 stallings per measurement run are detected on average. Thus, this number is much lower compared to the predefined bandwidth setting and shows that the player can adapt well towards changing bandwidth settings in real mobile networks.

An example measurement with a bandwidth setting of a real 3 G trace is plotted on the right side of Fig. 3. In the first 200 s, the bandwidth is changed according to the 3 G trace (1) while afterwards, 1.0 Mbit is set as bandwidth limit (2). It is shown that the complete bandwidth is required and used at the beginning of the stream to fill the buffer. The filling is slower compared to the constant bandwidth limits with 5.0 Mbit/s but works better than the filling in the variable predefined bandwidth example. However, the filling speed slows down at 75 s since the bandwidth drops from 1016 kbit/s to 650 kbit/s. Furthermore, it is shown that the buffer was filled before the bandwidth limitation behavior changes to a constant limit.

## Technical Validation

In order to ensure the technical validity of the collected data, the developed testbed and measurement procedure follows the guidelines of the DASH Industry Forum^25^. In doing so, we consider the recommendations for test cases for DASH-264/AVC HD and the recommendations for network emulation. In addition, the designed testbed relies on well-tested, peer-reviewed and freely available tools like the YouTube wrapper app^17^, which is freely available on Github^24^ or the state-of-the-art Linux command line tools *tc* and *tcpdump* to ensure consistent network emulation and data capture.

Finally, the dataset was extensively post-processed and cleaned to ensure all included measurement repetitions contain valid data points. This data cleaning contains the deletion of empty or erroneous measurements, invalid logs, and advertising or other cross-traffic as described in the data post-processing section and visualized in Fig. 2.

## Usage Notes

The complete dataset is available as zip file at figshare^23^. It contains all evaluation and post-processing scripts in the *materials* folder. The actual data representations are available in the *dataset* folder containing consecutively numbered subfolders. Each subfolder includes an *all_network_traffic.csv* file with the complete network and transport layer traffic, a *video_traffic.csv* file for the complete network and transport layer traffic of the video only, a *stats_for_nerds.csv* file with the complete application traffic during each measurement, a *application_data.csv* file with all application information for the video only, and a *bw_settings.csv* file with timestamps and bandwidth limitations for bandwidth changes.

The materials folder includes the following scripts: two evaluation scripts written in Python are added to simplify the work with the dataset. The *get_statistics.py* file reads all data and summarizes important information like video sizes, quality change and stalling information, buffer filling, and playtime data in the *data_overview.csv* file. Furthermore, the *plot_data.py* script requires the path to one measurement representation folder and whether TCP or UDP is used as transmission protocol. The script plots the bandwidth limit, the throughput, and the buffer filling status as shown in Fig. 3. Furthermore, it is possible for other researchers to extend the comprehensive dataset with our publicity available wrapper App approach^17^. Measurement results can then be evaluated with our post-processing scripts according to Fig. 2. The *process_stats_for_nerds.py* file receives the measured raw stats-for-nerds data and outputs a csv file for all application data in a readable format. Furthermore, the script *pcap_extraction.py* takes the raw pcap files including the complete network trace and exports a csv file with important information like timestamp, IP address, port, and packet payload size from the measurement. The script also extracts all video traffic together with its uplink requests as it is used in^36^. If only the video traffic should be extracted, the *video_only_traffic.py* separates video and cross-traffic. Since the dataset is extended by different streaming phases, the pseudocode *phase_detection_complete.pdf* describes the phase detection that extends the dataset together with an included table summarizing and explaining all relevant parameters in more detail. Please find the *readme.txt* in the dataset for additional explanation. In the following, further dataset usage possibilities are outlined to, for example, extend, understand, or verify related work.

Because of the popularity in public and the large data generation in current networks, streaming studies and analysis - especially with YouTube streaming - are an important and hot topic in research. The broad range of research topics with YouTube streaming include, among others, general network traffic and streaming studies for various applications^52,53^, streaming traffic separation^54,55^, streaming modeling and quality impairment detection^55,56^, and machine learning approaches to predict or assess streaming quality^49,57,58^. However, most of these works have one in common: the lack of a publicly available dataset. With this work, we close that gap and give other researchers the opportunity to improve and extend their research. Furthermore, with the presented dataset and the available application information together with the network and transport layer data, it is possible to model streaming behavior at different layers. The dataset improves potential packet level, request based, application layer studies. Furthermore, it is possible to study streaming traffic generation and behavior. With these insights, predictions of streaming impairments based on network and application data can be made. This can be used by streaming platforms or network operators to optimize data transmission, resource management, or general user satisfaction because of service improvement.

## Data Availability

The complete dataset is available as zip file at figshare^23^. The dataset includes all measured data and all post-processing and evaluation scripts. In addition, the publicly available wrapper app^17^ is freely available in case the dataset needs to be updated or expanded.
